# Supplementary material for: Axoneme polyglutamylation regulated by Joubert syndrome protein ARL13B controls ciliary targeting of signaling molecules
Source: Nat Commun. 2018 Aug 17;9:3310. doi: 10.1038/s41467-018-05867-1 (PMC6098020; doi:10.1038/s41467-018-05867-1)
Supplement: Supplementary file 1 — Supplementary Information [file 41467_2018_5867_MOESM1_ESM.pdf]

Axoneme polyglutamylation regulated by Joubert syndrome protein ARL13B controls ciliary targeting of signaling molecules

Kai et al.

Supplementary Information

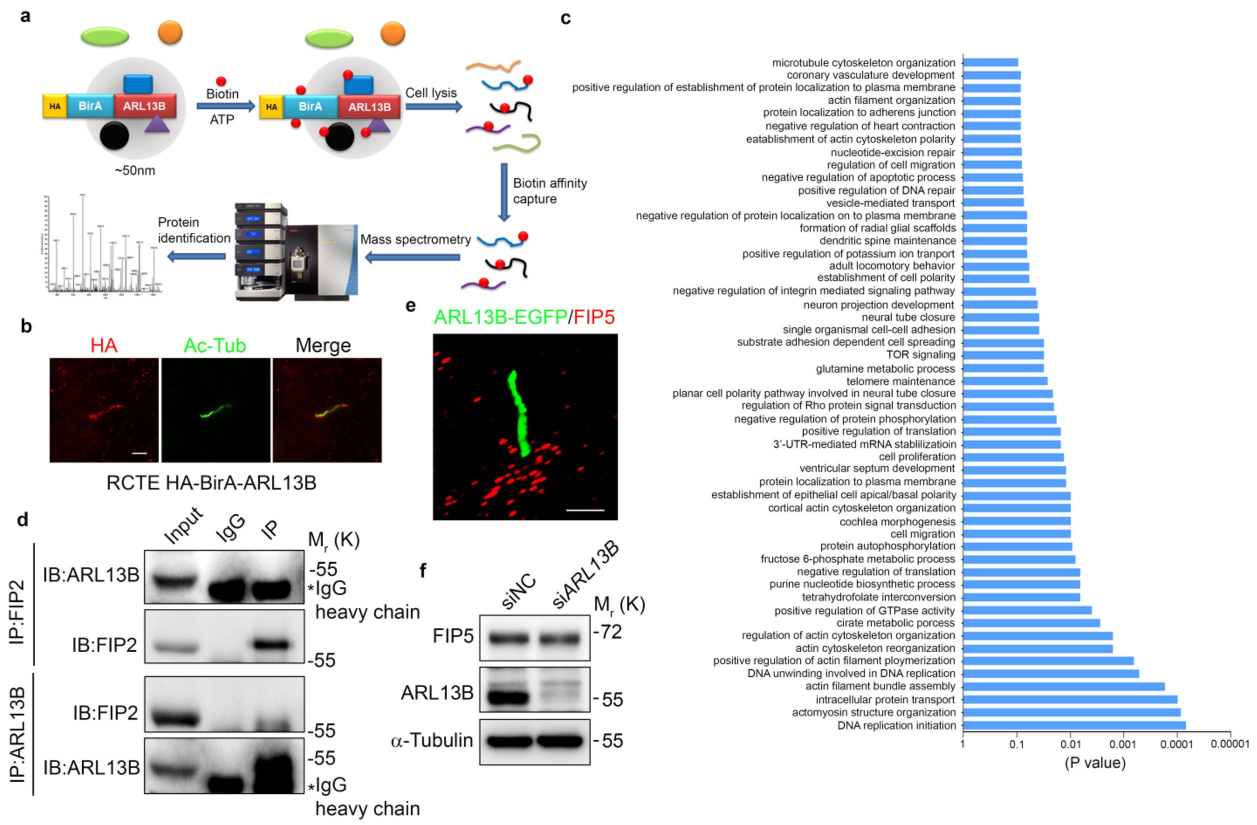

Supplementary Figure 1. ARL13B associates with FIP5. (**a**) Schematic model illustrating the work flow of Bio-ID experiment. (**b**) HA-BirA-ARL13B faithfully localizes in cilia in RCTE cells as seen by anti-HA antibody staining. (**c**) GO analysis of Bio-ID data. (**d**) In hTERT-RPE-1 cells, endogenous ARL13B does not associate with FIP2 during the early stage of ciliogenesis (4 h after serum starvation). (**e**) Structure illumination microscopic image of ARL13B-EGFP RCTE stable cells serum starved for 24 h. Images were processed by 3D transparency-rendering method. (**f**) Depletion of *ARL13B* does not affect expression levels of FIP5 as examined by western blotting. Scale bars: 2 μm.

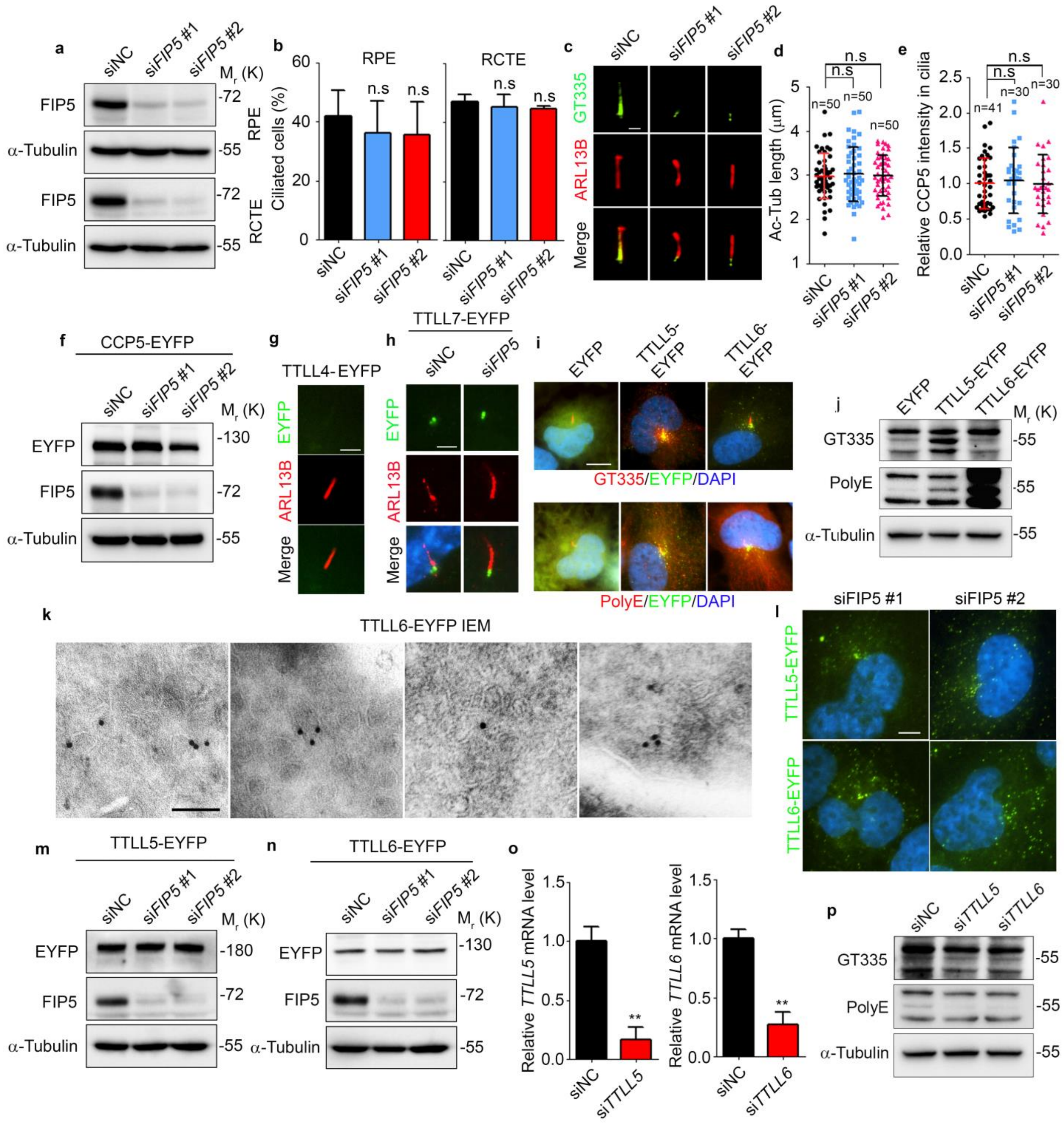

Supplementary Figure 2. ARL13B and FIP5 regulate axoneme polyglutamylation. (**a**) hTERT-RPE-1 and RCTE cells were treated with indicated siRNAs for 48 h. Efficiency of ARL13B siRNA was examined by western blotting. (**b**) Depletion of *FIP5* did not affect cilia formation as seen by examining the percentage of ciliated cells. (**c**) Depletion of *FIP5* totally abolished axoneme polyglutamylation in RCTE cells. (**d**) Depletion of *FIP5* did not affect axoneme acetylation in hTERT-RPE-1 cells as seen by measuring length of ciliary marker Ac-tub. N value: cilia number accessed from at least 6 fields. (**e**) Depletion of *FIP5* showed no effect on the ciliary level of CCP5 as examined by measuring ciliary intensity of CCP5-EYFP. **N** value: cilia number accessed from at least 6 fields. (**f**) Depletion of *FIP5* did not affect expression levels of CCP5-EYFP as examined by western blotting. (**g**) hTERT-RPE-1 stable cells

expressing TTLL4-EYFP cells were serum starved for 24 h. (**h**) hTERT-RPE-1 stable cells expressing TTLL7-EYFP were treated with indicated siRNAs and serum starved for 24 h. (**i, j**) Overexpression of TTLL5-EYFP and TTLL6-EYFP both induce hyperglutamylation of cytosolic microtubules but show different pattern in RPE cells in immunofluorescent (**i**) and western blotting (**j**). Overexpression of TTLL5-EYFP increases total polyglutamylation of the cytosolic microtubules as shown strong increase of both GT335 and PolyE signal, while overexpression of TTLL6-EYFP only increases long chain polyglutamylation as shown significant increase of PolyE but not GT335 signal. (**k**) Cryo-thin section of TTLL6-EYFP RPE cells were subjected to immunogold electron microscopy 24h after serum starvation. TTLL6-EYFP was immunostained by GFP antibody. The white circles indicate endosomal vesicles and black dots indicate TTLL6-EYFP. (**l**) The focal planes that show dispersed TTLLs-EYFP vesicles in indicated cells were shown by immunofluorescent. (**m, n**) Depletion of *FIP5* showed no effect on the expression level of TTLL5-EYFP or TTLL6-EYFP as examined by western blotting. (**o**) hTERT-RPE-1 cells were treated with indicated siRNAs for 48 h. Efficiencies of *TTLL5/6* siRNAs were examined by real-time PCR. Data in **b** and **n** are the statistical analysis of three independent experiments. **, $p<0.01$. **n.s.**, no statistically significant difference. Statistical significance was determined using unpaired student's t test. Center values represent mean. Error bars represent s.d. (**p**) Knockdown of *TTLL5* or *TTLL6* shows no significant decrease on overall polyglutamylation of cytosolic microtubules. Scale bars in **c**, **g** and **h** are 2 μm. Scale bar in **i** is 10 μm. Scale bar in **k** is 100 nm. Scale bar in **l** is 5 μm.

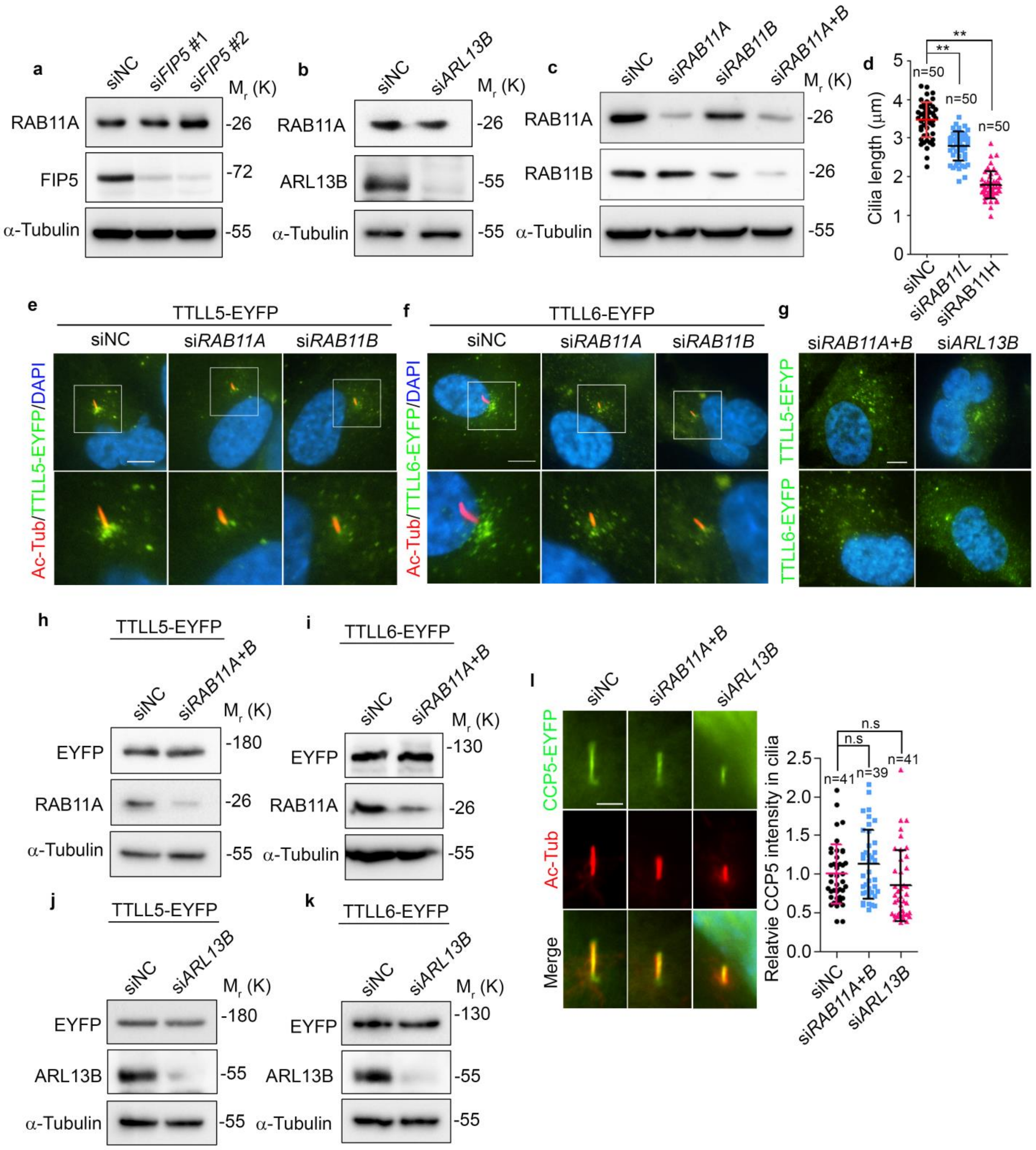

Supplementary Figure 3. RAB11 regulates axoneme polyglutamylation. (**a**) Depletion of *FIP5* did not affect the expression level of RAB11A as examined by western blotting. (**b**) Depletion of *ARL13B* did not affect the expression level of RAB11A as examined by western blotting. (**c**) hTERT-RPE-1 cells were treated with *RAB11A* or *RAB11B* siRNAs (20 nM) for 48 h. Efficiency of *RAB11* siRNAs was examined by western blotting. (**d**) hTERT-RPE-1 cells were treated with low (20 nM) or high (40 nM) dosage of *RAB11A+B* siRNAs for 48 h and serum starved for 24 h. Cilia length was quantified. **N** value: cilia number accessed from at least 6 fields. **, p<0.01. (**e**，**f**) hTERT-RPE-1 stable cells expressing TTLL5-EYFP (**e**) and TTLL6-

EYFP (**f**) were treated with 20 nM *RAB11A or RAB11B* siRNAs for 48 h and serum starved for 24 h, the impaired enrichment of TTLL5- or TTLL6-positive vesicles at the ciliary base was observed. Ac-Tub was immunostained by antibodies and TTLL5/6-EYFP was shown by direct fluorescence. (**g**) The focal planes that show dispersed TTLLs-EYFP vesicles in indicated cells were shown by immunofluorescent. (**h, i**) Depletion of *RAB11A+B* did not affect the expression level of TTLL5-EYFP or TTLL6-EYFP as examined by western blotting. (**j, k**) Depletion of *ARL13B* did not affect the expression level of TTLL5-EYFP or TTLL6-EYFP as examined by western blotting. (**l**) Depletion of *RAB11A+B or ARL13B* showed no effect on the ciliary level of CCP5 as examined by measuring ciliary mean intensity of CCP5-EYFP. Ac-Tub was immunostained by antibodies and CCP5-EYFP was shown by direct fluorescence. Statistical significance was determined using unpaired student's t test. Center values represent mean. Error bars represent s.d. Scale bars in **e-g** are 5 μm. Scale bar in **l** is 2 μm.

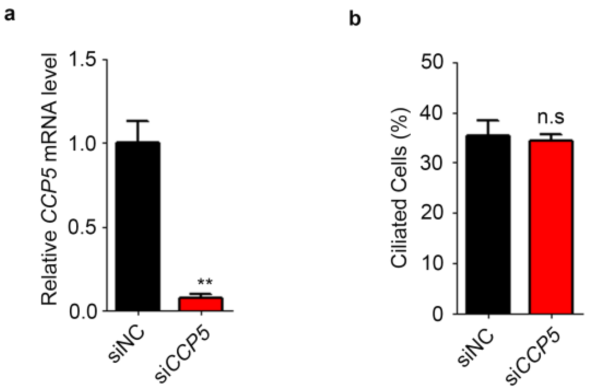

Supplementary Figure 4. Depletion of *CCP5* does not affect ciliogenesis. (**a**) hTERT-RPE-1 cells were treated with indicated siRNAs for 48 h. Efficiencies of *CCP5* siRNAs were examined by real-time PCR. **, p<0.01. (**b**) hTERT-RPE-1 cells were treated with indicated siRNAs for 48 h and serum starved for 24 h. Percentage of ciliated cells was quantified. Data are the statistical analysis of three independent experiments. **n.s**, no statistically significant difference. Statistical significance was determined using unpaired student's t test. Center values represent mean. Error bars represent s.d.

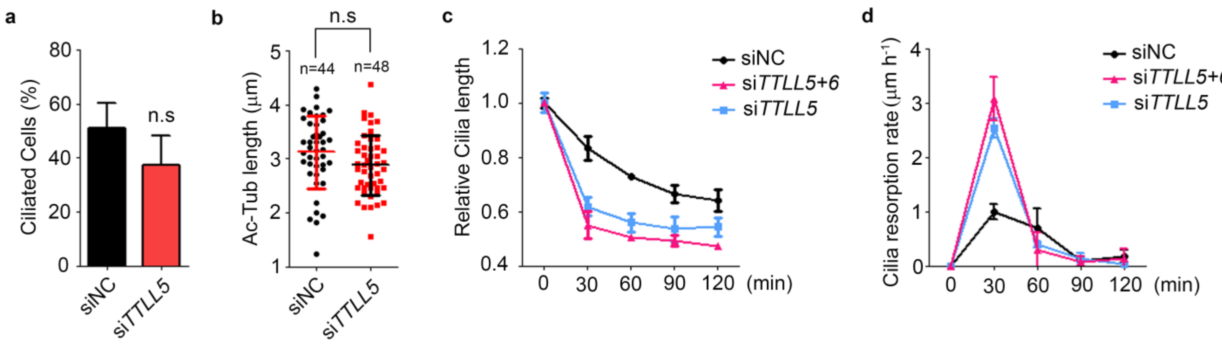

Supplementary Figure 5. Depletion of *TTLL5* does not affect ciliogenesis. (**a**) hTERT-RPE-1 cells were treated with *TTLL5* siRNAs for 48 h and serum starved for 24 h. Percentage of ciliated cells was quantified. Data are the statistical analysis of three independent experiments. (**b**) hTERT-RPE-1 cells were treated with *TTLL5* siRNAs for 48 h and serum starved for 24 h. The length of acetylated axoneme was measured and quantified. (**c, d**) hTERT-RPE-1 cells were serum starved for 24 h and then cultured in the medium containing 20% FBS for the indicated times. Relative cilia length (**c**) and resorption rate (**d**) were measured and quantified at the indicated times. **N** value: cilia number accessed from at least 6 fields. **n.s**, no statistically significant difference. Statistical significance was determined using unpaired student's t test. Center values represent mean. Error bars represent s.d.

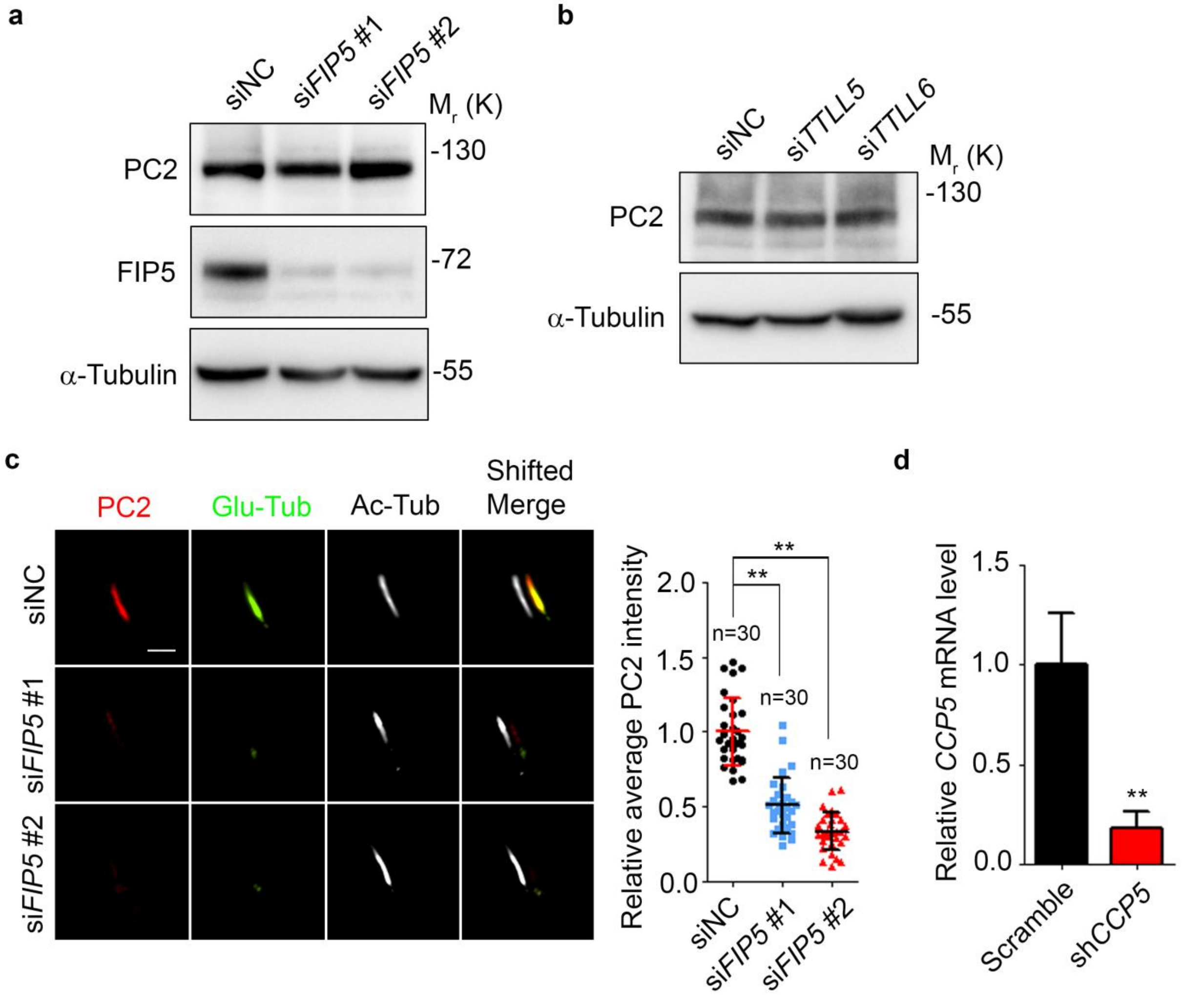

Supplementary Figure 6. The correlation between axoneme polyglutamylation and polycystin ciliary localization. (**a, b**) hTERT-RPE-1 cells were treated with indicated siRNAs for 48 h and serum starved for 24 h. The expression level of polycystin 2 (PC2) was examined by western blotting. (**c**) RCTE cells were treated with indicated siRNAs for 48 h and serum starved for 24 h and subjected to immunofluorescent staining. The relative intensity/μm^2 of ciliary PC2 was quantified in the right panel. In *FIP5*-depleted cells, hypoglutamylated cilia were measured for quantifying the intensity/μm^2 of ciliary PC2. Scale bar: 2 μm **N** value: cilia number accessed from at least 6 fields. (**d**) hTERT-RPE-1 cells were treated with the indicated shRNAs using lentivirus system. Efficiency of shRNA targeting *CCP5* was examined by real-time PCR. Data are the statistical analysis of three independent experiments. Statistical significance was determined using unpaired student's t test. **, p<0.01. Center values represent mean. Error bars represent s.d.

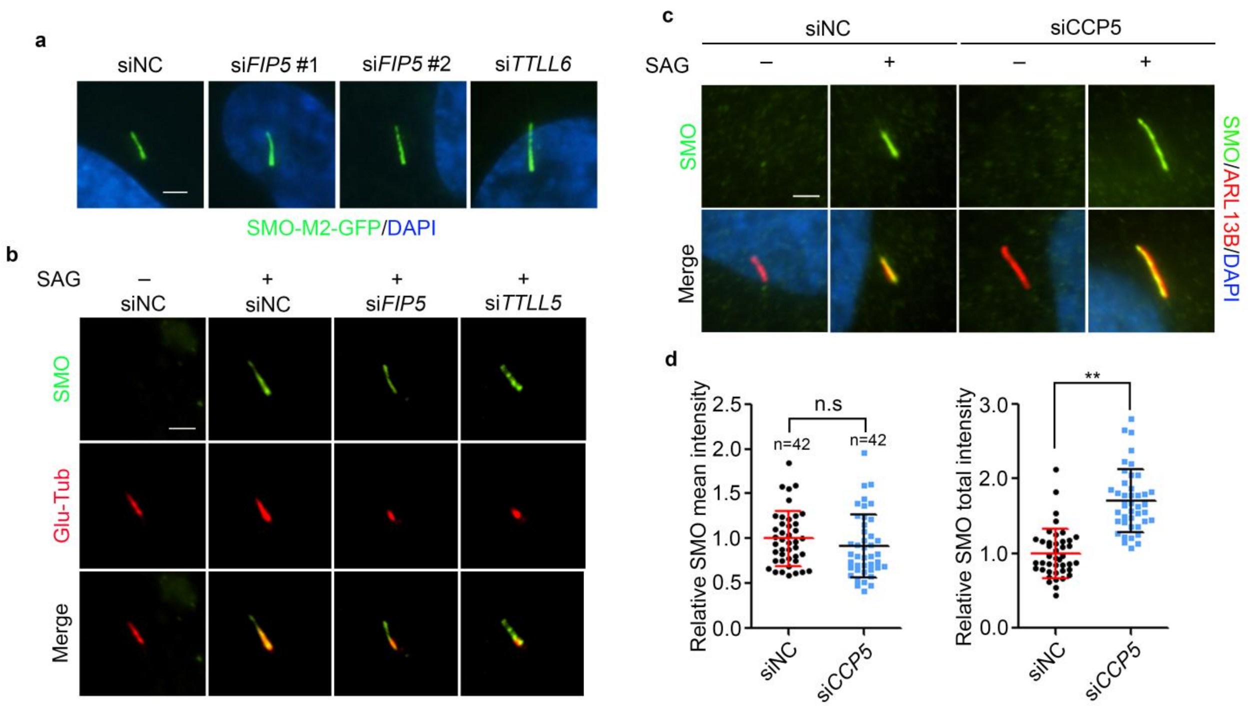

Supplementary Figure 7. Axoneme hypoglutamylation does not affect SMO ciliary translocation. (**a**) SMO-M2-GFP expressed hTERT-RPE-1 stable cells were treated with indicated siRNAs for 48 h and serum starved for 24 h. Ciliary localizations of SMO-M2-GFP were examined. (**b**) hTERT-RPE-1 cells were treated with the indicated siRNAs for 48 h, serum starved and treated with 500 nM SAG for 24 h. Ciliary localizations of SMO were examined. (**c, d**) hTERT-RPE-1 cells were treated with the *CCP5* siRNA for 48 h, serum starved and treated with 500 nM SAG for 24 h. Ciliary localizations of SMO were examined by immunofluorescent assay (**c**), and the mean or total intensity of ciliary SMO was quantified (**d**). Statistical significance was determined using unpaired student's t test. Center values represent mean. Error bars represent s.d. Scale bars: 2 μm.

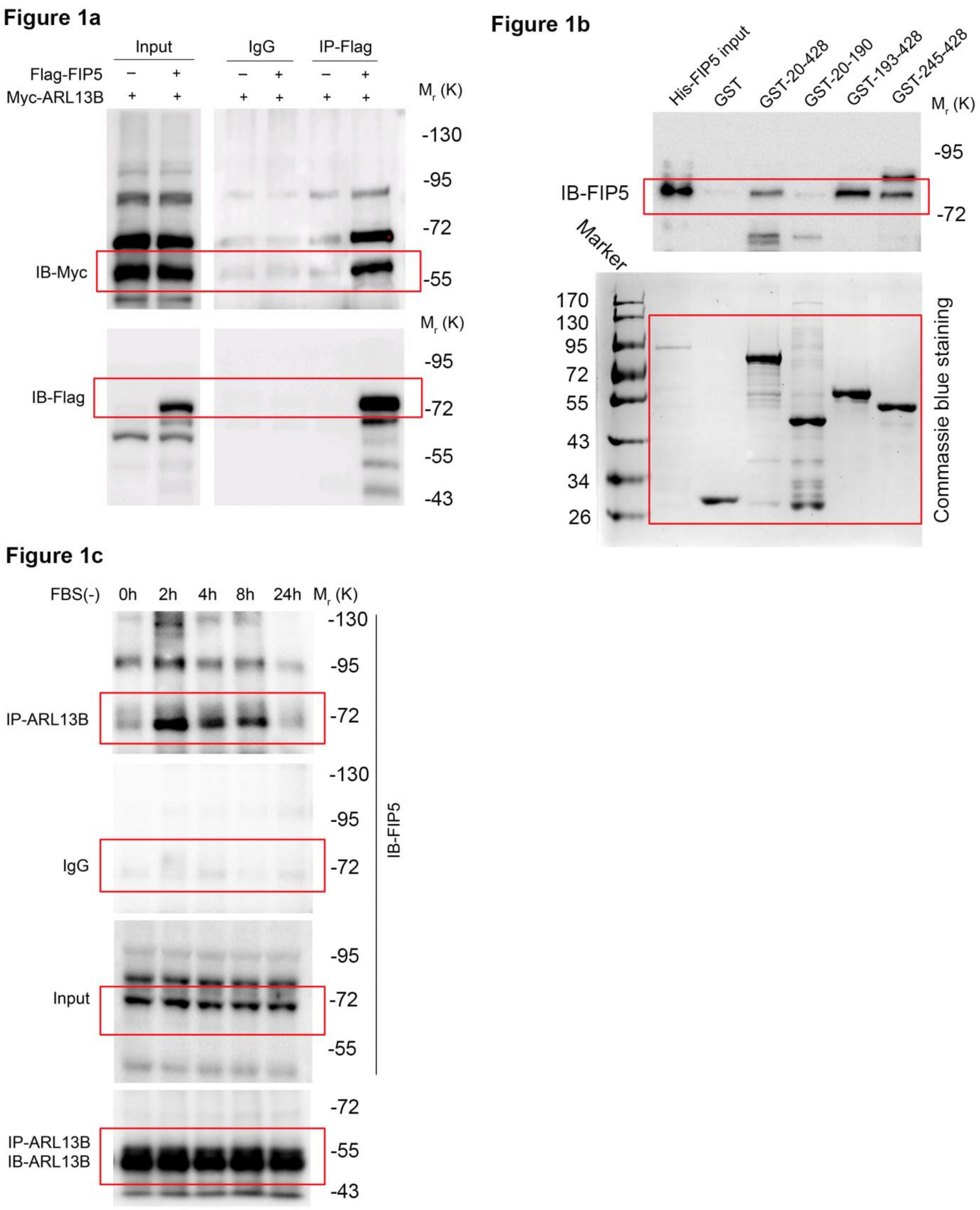

Supplementary Figure 8. Uncropped scans of blots shown in Figure 1a, 1b and 1c.
