# Supplementary material for: Axoneme polyglutamylation regulated by Joubert syndrome protein ARL13B controls ciliary targeting of signaling molecules
Source: Nat Commun. 2018 Aug 17;9:3310. doi: 10.1038/s41467-018-05867-1 (PMC6098020; doi:10.1038/s41467-018-05867-1)
Supplement: Supplementary file 3 — Description of Additional Supplementary Files [file 41467_2018_5867_MOESM3_ESM.pdf]

Description of Additional Supplementary Files

File Name: **Supplementary Movie 1**
Description: **FIP5-positive vesicles show immediate enrichment upon serum starvation.** Live-cell time-lapse imaging of FIP5-mCherry expressing RPE cells were performed every 10 min during the first 6 h upon serum withdrawal.

File Name: **Supplementary Movie 2**
Description: **FIP5-positive vesicles associate with ARL13B compartment during early stage of ciliogenesis.** At 2h after serum starvation**,** 3D structure of ARL13B-EGFP (Green) and FIP5-positive vesicles (Red) in RCTE cells was obtained by SIM microscopy with transparency-rendering method.

File Name: **Supplementary Movie 3**
Description: **FIP5-positive vesicles do not associate with ARL13B compartment in mature cilia.** At 24h after serum starvation**,** 3D structure of ARL13B-EGFP (Green) and FIP5-positive vesicles (Red) in RCTE cells was obtained by SIM microscopy with transparency-rendering method.

File Name: **Supplementary Movie 4**
Description: **FIP5-positive vesicles localize immediately adjacent to TTLL5-positive vesicles at cilia base.** At 24h after serum starvation**,** 3D structure of Ac-Tub (White), TTLL5-EYFP vesicles (Green) and FIP5-positive vesicles (Red) in RPE cells was obtained by SIM microscopy with transparency-rendering method.

File Name: **Supplementary Movie 5**
Description: **FIP5-positive vesicles localize immediately adjacent to TTLL6-positive vesicles at cilia base.** At 24h after serum starvation**,** 3D structure of Ac-Tub (White), TTLL6-EYFP vesicles (Green) and FIP5-positive vesicles (Red) in RPE cells was obtained by SIM microscopy with transparency-rendering method.

File Name: **Supplementary Data 1**
Description: **List of Bio-ID candidate proteins.** Identified Proteins in Bio-ID proteomic mapping of human ARL13B interactors are listed in this table. Candidates functioning in trafficking, GTPase regulation, and cytoskeleton organization, known ciliary proteins (IFT74, TULP3, KIF5B, etc.) and ARL13B interactors (such as INPP5E, ARL3, Myh9, etc.) are highlighted in the list.
